# Can Chromoendoscopy Improve the Early Diagnosis of Gastric Carcinoma in Dogs?

**DOI:** 10.3390/ani12172253

**Published:** 2022-08-31

**Authors:** Marcus Vinicius Candido, Pernilla Syrjä, Susanne Kilpinen, Søren Meisner, Mohsen Hanifeh, Thomas Spillmann

**Affiliations:** 1 Department of Equine and Small Animal Medicine, Faculty of Veterinary Medicine, P.O. Box 57, Helsinki University, 00014 Helsinki, Finland; 2 Department of Veterinary Biosciences, Veterinary Pathology, Faculty of Veterinary Medicine, P.O. Box 66, Helsinki University, 00014 Helsinki, Finland; 3 Digestive Disease Center, Bispebjerg Hospital, University of Copenhagen, 2400 Copenhagen, Denmark

**Keywords:** dog, gastric carcinoma, mucous metaplasia, glandular dysplasia, endoscopy, chromoendoscopy, narrow band imaging, indigo carmine, cancer

## Abstract

**Simple Summary:**

Currently, canine gastric carcinoma is mainly diagnosed in its late, incurable phase, and strategies for early diagnosis are lacking. In human medicine, chromoendoscopic (CE) methods such as staining the gastric mucosal surface with indigo carmine (IC), and narrow band imaging (NBI), have improved the diagnosis of precancerous gastric mucosal changes and early gastric carcinoma. This study aimed at investigating whether IC-CE and NBI-CE can improve the diagnostic yield of endoscopy in dogs. Belgian Shepherd dogs are predisposed to gastric carcinoma; thus, 30 dogs of the breed served as the study population. As a result, the study revealed that especially the combination of standard white light endoscopy (WLE) with NBI-CE allows better recognition of gastric mucosal structural changes than WLE alone. However, CE assessment templates used to predict the type of mucosal change in humans were not applicable in dogs. The value of the study lies in providing evidence that CE can improve the diagnosis of precancerous changes and early gastric carcinoma in dogs. However, current image assessment templates from human medicine need major adjustments to comprehend canine gastric mucosal conditions.

**Abstract:**

Chromoendoscopy has improved the early diagnosis of gastric cancer in humans but its usefulness in dogs is unknown. This study aimed at assessing whether adding narrow band imaging (NBI) or indigo carmine (IC) chromoendoscopy (CE) can improve the diagnostic yield of standard white light endoscopy (WLE). We compared the real-time findings of canine WLE, NBI-CE, and IC-CE and corresponding histology reports with endoscopic mucosal pattern assessment templates used in human medicine. Belgian Shepherd dogs are predisposed to gastric carcinoma. Therefore, 30 dogs of this breed served as the study population. According to histology, 17/30 dogs had mucosal changes (mucous metaplasia, glandular dysplasia, and gastric carcinoma). Diagnostic yield was best when targeted biopsies were taken with WLE and NBI-CE combined (15/17 cases). WLE alone positively identified only 8/17 cases and missed a gastric carcinoma in 3/6 cases. CE assessment templates based on macroscopic mucosal patterns, broadly used in human medicine, were not readily applicable in dogs. In conclusion, the study provides evidence that using CE in dogs has the potential to improve the diagnosis of precancerous gastric mucosal pathology and early gastric carcinoma. However, current image assessment templates from human medicine need major adjustments to the patterns of canine gastric mucosa.

## 1. Introduction

Canine gastric carcinoma is the most common gastric tumor in dogs older than 8–10 years. Currently, the tumor is diagnosed mostly at an advanced stage with a 70–90% rate of metastasis [1]. Non-polypoid tumors have a rate of invasion and metastasis of 100% and polypoid tumors of 21% [2]. Treatment of choice is tumor resection with wide margins and removal of local lymph nodes, but the median survival time after surgery (178 days; range 1–1902 days) is rather short and depends on the rate of local invasion and metastasis [3]. Current research looks for possibilities to advance the early diagnosis of gastric carcinoma, to improve the therapeutic outcome.

Gastroscopy and mucosal biopsy sampling for histology are established as the standard procedure to detect and differentiate gastric mucosal pathology in dogs [4]. In humans, gross mucosal changes are easily identified under standard white light endoscopy (WLE), but more subtle, early changes may be overlooked, and random biopsy might fail to sample from pathologically changed mucosa, leading to possible misdiagnosis [5]. Recent endoscopic studies in the Belgian Shepherd dog breed types Tervuren and Groenendael revealed an association of gastric mucosal structural changes such as mucous metaplasia and glandular dysplasia with gastric carcinoma. Like in humans, these pathological gastric mucosal changes should be considered preneoplastic. Overlooking those changes when using WLE could have harmful consequences for the patient [6]. Attempts in human medicine to overcome the limitations of WLE in visualizing subtle macroscopic mucosal changes have led to the development of chromoendoscopy (CE). CE allows an enhancement of mucosal surface patterns, color, and vascularization, inducing a paradigm shift from random to targeted biopsies in patients with subtle gastric mucosal changes [7]. Examples of such novel imaging technologies are narrow band imaging (NBI), Storz professional image enhancement system (SPIES), and flexible spectral imaging color enhancement, all using specifically developed endoscopy equipment [8,9,10]. Studies in humans revealed that, for example, NBI allows the identification of gastric mucosal patterns with high predictive value for the final histologic diagnosis of normal mucosa (83%), intestinal metaplasia (84%), and dysplasia (95%) [11]. To achieve such image improvement, NBI technology uses the wavelengths of blue and green light and further digital processing.

Another, and less expensive way to improve the visualization of gastric mucosal structures, allowing for targeted instead of random biopsies in humans, has been the use of in-vivo staining methods, by spraying dyes on the mucosal surface during standard WLE [12]. Indigo carmine (IC) works by enhancing the contours of mucosal groves and has been one of the more commonly used CE dyes. IC-CE increases the detection of dysplastic lesions in humans by three to five-fold as compared to using WLE alone [13].

In veterinary gastroenterology, the possibilities to detect subtle changes through targeted biopsies have been limited to the use of WLE. It remains rather challenging to predict the possible mucosal pathology and to detect preneoplastic processes and malignancies early and accurately, especially when they are still subtle and confined to the gastric mucosa [1]. Despite the increased availability of CE equipment in veterinary medicine, there have been only very few studies exploring the possibilities of CE for canine and feline patients [9,14]. It remains so far uncertain whether CE techniques can improve the diagnostic yield of endoscopy in dogs.

This study was designed to assess the possibilities of WLE, NBI-CE, and IC-CE in visualizing gastric mucosal structural changes such as mucous metaplasia, glandular dysplasia, and gastric carcinoma in dogs. For this, we compared histologic reports with real-time gastroscopy findings, and images saved using WLE and two CE methods taken during a prospective clinical trial in Belgian Shepherd dogs. The primary objective of this study was to assess whether real-time findings noted during examination with WLE, NBI-CE, and IC-CE, or a template-based interpretation of recorded images of mucosal patterns in NBI-CE, or IC-CE images, can predict the histologically confirmed presence of mucosal changes of concern (mucous metaplasia, glandular dysplasia, and gastric carcinoma). The second objective was to assess whether targeted biopsies directed by WLE in combination with NBI-CE or IC-CE have a better diagnostic yield at histology than an approach using non-targeted biopsies during WLE alone.

## 2. Materials and Methods

### 2.1. Study Population and Ethical Licensing

WLE, NBI-CE, and IC-CE of the stomach were performed successively during the same endoscopic procedure in 30 Belgian Shepherd dogs. Here, 19 Tervuren, eight Groenendael, and three Malinois attended a prospective clinical trial on the diagnosis of gastric mucosal changes, namely mucous metaplasia, glandular dysplasia, and carcinoma [3]. Prior to endoscopy, all dogs fasted for 12–16 h, then received orally a mixture of 20% acetylcysteine (3 mL, Aurum Pharmaceuticals Ltd., Essex, UK) and 0.526% simethicone (15 mL, Endoparactol, Hormosan Pharma GmbH, Frankfurt am Main, Germany) to improve the visibility of the gastric mucosa [15]. Endoscopy was performed under general anesthesia with two different anesthesia protocols as previously described [16]. WLE, NBI-CE, and IC-CE images were saved in a local image bank during the same study for an external blinded assessment by a medical doctor experienced in gastroscopy in humans (S.M.). The study protocol was approved by the Viikki Campus Research Ethics Committee, University of Helsinki (Statement 5/2015).

### 2.2. Endoscopic Equipment and Image Acquisition

WLE and NBI-CE were performed successively by experienced endoscopists (M.C., T.S.) with a first-generation NBI 180 system (Olympus Europa SE & Co. KG, Hamburg, Germany) consisting of an endoscope (CF180AL), light source (CLV-180), image processor (CV-180), carbon dioxide insufflation unit (UCR), and an irrigation pump (OFP-2) for washing debris from the gastric mucosa during the endoscopy.

IC-CE was performed right after NBI-CE with the same equipment but under white light and by inserting a spray catheter (PW-5V-1, Olympus Europe) through the endoscope’s working channel. IC stock solution (indigo carmine 1%, Life partners Europe, Bagnolet, France) was diluted in sterile water to a 0.2% working solution. To avoid excessive dye accumulation, the working solution was sprayed sparingly onto the entire gastric mucosal surface.

**Figure 1:**
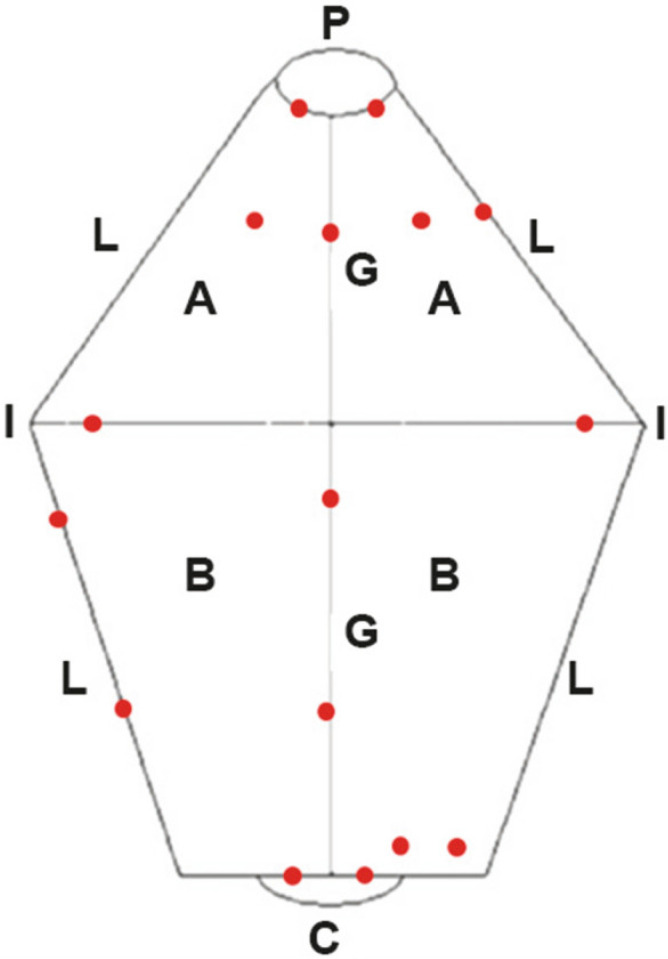
Schematic map of the stomach showing standard biopsy sites (red dots) used for non-targeted gastric mucosal sampling (modified from Simone et al. [17]). A: antrum; B: gastric body; G: greater curvature; L: lesser curvature; P: pylorus; I: incisura angularis (angular fold); C: cardia.

During the WLE, NBI-CE, and IC-CE procedures, images were taken at full gastric insufflation from standardized viewpoints and captured with MediCap USB200 (MediCapture Inc., Plymouth Meeting, PA, USA). The files were finally stored in the Microsoft Outlook cloud of the University of Helsinki, Finland. The standard viewpoints for taking images during WLE, NBI-CE, and IC-CE were (1) gastric body, (2) fundus and cardia, (3) angular fold (incisura) with fundus and antrum (two chamber view), and (4) pylorus. The gastric mucosa was then examined closely in search of diffuse or focal changes. The location and distribution of changes were recorded when the pictures were taken, and were numbered in a schematic gastric map (Figure 1). The changes were listed in a numbered table along with a short macroscopic description.

### 2.3. Targeted and Non-Targeted Biopsy Sampling and Histologic Assessment of Gastric Mucosal Microstructure

For histology, two separate sets of gastric mucosal samples were taken to compare the diagnostic outcome of biopsies as indicated by WLE alone, or by the combination of WLE and CE. When the endoscopic appearance was unremarkable, a non-targeted biopsy routine was applied. In short, two samples each were taken from cardia, fundus, greater and lesser curvature of the gastric body, incisura, and pylorus, and one from each quadrant of the antrum (clockwise at 12, 3, 6, and 9 o’clock) (Figure 1).

*Biopsy Set 1* included targeted biopsies from the sites presenting mucosal changes visible after the three subsequent endoscopic techniques: (1) WLE, (2) NBI-CE, and (3) IC-CE. The samples were identified according to the gastric region, or a short description of the targeted change (e.g., ulcer, mass, unusual texture). In the absence of visible mucosal changes, non-targeted sampling was applied (Figure 1). After that, the second set of samples *(Biopsy Set 2)* was taken either from the predefined locations shown in Figure 1, or from the mucosal changes recorded in those regions during WLE, and labeled according to the gastric region sampled.

All mucosal samples were collected with disposable biopsy forceps (FB-210U, Olympus Europe) and placed onto wood chips, then immersed (upside-down) in 10% formalin for fixation, in separate vials. Each vial was labeled as part of the Biopsy Set 1 or 2, as well as the gastric region, and/or respective change (Biopsy Set 1). Therefore, at least four separate vials (and up to 6 in Biopsy Set 1), were used for the gastric regions (1) cardia + fundus, (2) body, (3) incisura, and (4) antrum + pylorus. All biopsy specimens were collected after IC dye-spraying and IC-CE image recording to avoid interference of blood from biopsy sites with the IC-CE imaging.

For histology, the labeled samples were paraffin-embedded and cut into sections of 4 μm. The sections were routinely stained with hematoxylin-eosin, but special staining techniques such as periodic acid-Schiff reaction were applied in selected cases at the pathologist’s discretion. Immuno-histochemical staining for epithelial cells was performed with anti-cytokeratin AE1/AE antibodies (anti-human Ck AE1/AE3, mouse monoclonal M3515, Dako Agilent, Santa Clara, CA, USA). Antigens were retrieved with 0.01 M citrate buffer at pH 6 and heated for 20 min at 99 °C, then revealed following the instructions for UltraVision Detection System HRP/DAB kit (Thermo Fisher Scientific, Waltham, MA, USA). Biopsy Set 1 was used for the diagnostic workup, and Biopsy Set 2 was examined in a blinded fashion at the end of the study for comparison of results from biopsies directed by the combination of WLE and CE, or by WLE alone.

The histological slides of both sample sets were examined according to recommendations by the World Small Animal Veterinary Association’s International Gastrointestinal Standardization Group [18]. The slides were also examined for histopathologic structural changes, including mucous metaplasia, glandular dysplasia, and type of gastric carcinoma [19,20]. The presence and severity of mucosal inflammation (intraepithelial lymphocytes, and lymphoplasmacytic, eosinophilic or neutrophilic infiltration into lamina propria, was scored as normal = 0, mild = 1, moderate = 2 or severe = 3 [21].

### 2.4. Real-Time Assessment of Endoscopic Findings during WLE, NBI-CE, and IC-CE

Every endoscopic procedure started with standard WLE, and the gastric mucosa was subjectively assessed in real time as unremarkable, diffusely changed, or focally changed. Diffuse and focal changes were described in the patient record and drawn in a printout of a schematic map of the stomach (Figure 1) that was added to the patient’s records. In addition, standard images were taken as described above. Once this examination was finalized, the NBI setting of the processor was activated and the endoscopic examination was repeated in the same fashion as for WLE. Gastric mucosal changes that differed from WLE findings (e.g., focal changes) were recorded in the same fashion as for WLE. As last, IC-CE was performed by spraying IC on the gastric mucosa surface and the same assessment steps were repeated.

Results of the real-time assessment of WLE, NBI-CE, and IC-CE findings were compared with the histological findings of targeted and non-targeted biopsies to estimate the sensitivity, specificity, and accuracy of every single endoscopic method, or the combination of WLE with NBI-CE and IC-CE for predicting mucous metaplasia, glandular dysplasia, and gastric carcinoma.

### 2.5. Template-Based NBI-CE and IC-CE Image Assessment of Gastric Mucosal Surface Patterns

During each endoscopic examination, two separate sets of images were captured, first using NBI-CE and then IC-CE. The images saved from each patient were reviewed by the endoscopist (M.C.) to select a subset of images that corresponded to localizations that had also been biopsied for histology. Among the images available, those with the best quality (focus, resolution, color) were selected. The images included focal changes, as well as pictures taken from standard viewpoints in case no macroscopic changes were seen in a dog during NBI- or IC-CE. The files were named according to the localization of the image (e.g., pylorus, body distal greater curvature, etc.). These digital images were submitted for evaluation by a human gastroenterologist experienced in NBI- and IC-CE (S.M.). The sets of images taken with NBI-CE and with IC-CE were sent in separate lots two months apart to minimize possible identification of the cases by recollection. The external examiner was unaware of the histopathological diagnoses.

To evaluate the NBI-CE images, the examiner applied the template validated and widely used in humans to identify changes related to intestinal metaplasia, dysplasia, and carcinoma. The original template comprises standard attributes for mucosal pattern, mucosal color, and vascular pattern [22].

The mucosal patterns detected in NBI-CE images of the dogs were classified either as normal round, normal mosaic, and polygonal, enlarged mosaic and polygonal, or tubulo-villous (Table 1), conforming to the template applied in human gastroenterology.

For the assessment of IC-CE images, the mucosal patterns were graded as normal or suspicious according to the examiner’s (S.M.) expertise in human gastroscopy because there have been no templates for IC-CE assessment in human medicine. Examples of such categories are shown in Table 2.

### 2.6. Statistical Analysis

Descriptive statistics were used to assess the ability of WLE, NBI-CE, and IC-CE to select the biopsy site with the most severe gastric mucosal changes.

NBI-CE images graded as normal mucosal patterns (normal round, mosaic, and polygonal) were regarded as suggesting the absence of disease, thus were counted and analyzed as negative test results. Abnormal patterns (enlarged mosaic and tubulo-villous) were regarded as indicating the presence of gastric mucosal pathology (positive for metaplasia, dysplasia, or carcinoma). For IC-CE, images suggesting normal mucosal structure were assessed as negative for gastric mucosal structural pathology. The images regarded as suspicious for gastric mucosal pathology were assessed as positive. The ability of NBI-CE or IC-CE to predict gastric mucosal changes was assessed by calculating sensitivity, specificity, and diagnostic accuracy.

## 3. Results

### 3.1. Dogs and Endoscopic Findings

Endoscopic examinations were performed in 30 Belgian Shepherd dogs belonging to the breed varieties Tervuren (*n* = 19), Groenendael (*n* = 8), and Malinois (*n* = 3). Patient signalment and the diagnostically most important endoscopic findings of the gastric mucosa are summarized in Table 3. Based on real-time endoscopic assessment, seven dogs had unremarkable gastric mucosa and 23 dogs had changes that were diffuse in 14 dogs, focal (non-ulcerative) in three dogs, and ulcerative in six dogs.

**Figure 2:**
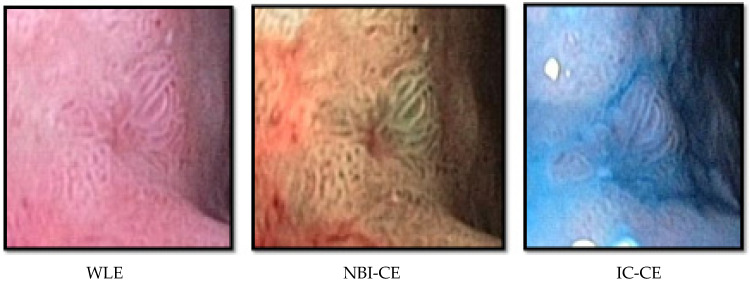
Sample images of the same focal change in Dog 6, as seen at regular white light endoscopy (WLE), narrow band imaging chromoendoscopy (NBI-CE) and indigo carmine chromoendoscopy (IC-CE). Histology revealed mucous metaplasia and glandular dysplasia.

Conformity between the real-time findings from WLE and the expert assessments of still images taken during NBI- and IC-CE (as illustrated in Figure 2) was overall moderate. Regarding the presence (positive) or absence of changes of concern (negative), WLE and NBI-CE agreed in 18/30 dogs and disagreed in 12/30 dogs. Concordance between WLE and IC-CE was seen in 26/30 dogs and disparity in only 4/30. The blinded expert assessments of NBI- and IC-CE images were compatible in 18/30 and divergent in 12/30 dogs.

### 3.2. Histology of Targeted and Non-Targeted Gastric Mucosal Biopsies

Histology was performed in two sample sets. Biopsy Set 1 contained biopsies directed by the combination of WLE and CE. Biopsy Set 2 comprised biopsies taken as dictated by WLE alone. The results of Biopsy Set 1 were used for diagnostic purposes and assessed immediately. Biopsy Set 2 was withheld after processing: the histologic slides were assessed in a blinded fashion at the end of the study and their results were compared with those of the targeted biopsies.

Targeted biopsies were taken from the focal or diffuse changes visible in WLE, NBI-CE, and/or IC-CE. However, in 5/30 dogs (Dogs 18, 20, 26, 27, 30) no obvious changes were seen in WLE, NBI-CE, or IC-CE. In these dogs, non-targeted biopsies were taken for both sample sets from predefined locations as presented in Figure 1.

Based on the histologic assessment of biopsy set 1 (CE-directed, mostly targeted biopsies), none of the dogs showed entirely normal gastric mucosa and the diagnostically most important histologic findings were mucosal inflammation of different types in 30/30 dogs (Table 3). While 16/30 dogs displayed submucosal and mucosal inflammation of different severity, 15/30 dogs had also mucosal structural changes such as mucous metaplasia (*n* = 8), glandular dysplasia (*n* =13), and/or gastric carcinoma (*n* = 6). As summarized in Table 3, the parallel occurrence of two or more types of mucosal change was seen in 9/15 dogs. Three dogs had concurrent mucous metaplasia, glandular dysplasia, and carcinoma (Dogs 1, 6, 15); three had dysplasia and carcinoma (Dogs 17, 23, 29); and the other three (Dogs 22, 24, 25) had metaplasia and dysplasia. One of them (Dog 24) was diagnosed with gastric carcinoma one year later [6].

When comparing the most severe histological changes detected in biopsy set 1 with those in biopsy set 2 of individual dogs, a complete agreement was seen in 18/30 dogs but disagreements in 12/30 dogs (Table 3). From the disagreements in 12 dogs, biopsy set 1 revealed more severe mucosal structural changes than biopsy set 2 in 9/12 dogs. Misdiagnosis based on non-targeted biopsies occurred in three of the dogs with carcinoma (one misdiagnosed as mucous metaplasia (dog 1) and two misdiagnosed as inflammatory (dogs 15, 29)), and in three dogs with glandular dysplasia (two misdiagnosed as inflammation (dogs 11, 16) and one as mucous metaplasia (dog 22)). Dog 11 was the only patient where intestinal, but not mucous, metaplasia was reported from non-targeted, set-2 biopsies. In three dogs (dogs 15, 24, 25), mucous metaplasia was seen in targeted (set 1) but not in non-targeted (set 2) biopsies. However, there were 2/12 dogs with disagreeing results in which non-targeted Set-2 biopsies revealed glandular dysplasia but set-1 biopsies were regarded as inflammatory without mucosal structural changes (dogs 5, 10). In dog 23, glandular dysplasia and gastric carcinoma were confirmed in both biopsy sets but only non-targeted biopsies revealed mucous metaplasia. In all five dogs that had non-targeted biopsies at both biopsy sets (dogs 18, 20, 26, 27, 30), the histology findings agreed.

Combining both sample sets revealed no mucosal changes in 13/30 dogs, but 17/30 dogs had either mucous metaplasia, glandular dysplasia, or gastric carcinoma as the most severe histologic finding. From 17 dogs with mucosal pathology, nine were diagnosed in targeted biopsies only, six in both targeted and non-targeted biopsies, and two in non-targeted biopsies only. When linking histology with the endoscopic methods, the diagnostic yield of mucosal structural pathology was best when targeted biopsies were taken under WLE combined with NBI-CE (15/17 cases). WLE alone detected these lesions only in 8/17 cases and missed a gastric carcinoma in 3/6 cases (50%).

### 3.3. Template-Based Assessment of Images Taken during NBI-CE

For an independent image assessment, 143 NBI-CE images were selected and sent to the external evaluator (S.M.), who was blinded to the histologic findings. Of these, 27 images were used only for anatomical orientation and were not included in the template-based assessment. Seventeen other images had insufficient image quality and another 12 images that failed to match the template were excluded by the examiner. Thus, a total of 87 NBI-CE images were assessed according to the given NBI-CE assessment (Table 4). However, it was not possible to apply the human NBI template for assessing mucosal color and vascular pattern to the gastric mucosa of dogs, because the images neither showed color changes typical for gastric pathologies in humans nor were the vascular patterns relatable.

The mucosal patterns of 12 NBI-CE images of 10 dogs that did not match the template were therefore reported separately. Of these images, three were assessed as representing active or healing ulcers, one of which was not confirmed by histology (dog 3). The other two were confirmedly ulcerative, with one being inflammatory (dog 24) and the other an adenocarcinoma of intestinal type (dog 23). Dog 23 also showed marked differences between two types of gastric mucosa with bluish color, which was however not confirmed as intestinal metaplasia, as described in humans [23]. Small hyperplastic areas were suspected in two dogs, of which histology revealed normal mucosa with mild acute hemorrhage in one case (dog 10) and, in the other case, moderate lymphoplasmacytic infiltration, multifocal hyperemia, mild hemorrhage, fibrosis in the lamina propria, and mild superficial edema (dog 9). In two dogs, the mucosa was assessed as normal despite differing from the template (dogs 20 and 30). Both dogs had chronic diffuse gastritis with an eosinophilic component and different degrees of diffuse or multifocal fibrosis. In two dogs, the endoscopic appearance suggested local blood flow disorders such as ischemia (dog 11) or hypervascularity (dog 25). While dog 11 had histologically a mild diffuse chronic eosinophilic gastritis with mild fibrosis and mucous metaplasia in the angular fold, dog 25 had a severe diffuse chronic atrophic gastritis, with mild multifocal eosinophilic and neutrophilic components. In dog 16, a small adenoma in the pylorus area was suspected. Histology revealed intact epithelium and diffuse severe lymphoplasmacytic infiltration with a moderate neutrophilic and eosinophilic component. The gland structure was in part moderately thinned and replaced by fibrosis.

The application of the human template for the evaluation of gastric NBI-CE images as normal (negative) or abnormal (positive) showed a sensitivity of 39%, a specificity of 82% and a diagnostic accuracy of 67%.

### 3.4. Template-Based Assessment of Images Taken during IC-CE

When the IC dye was sprayed on the gastric mucosa during IC-CE, the perception of surface details of the mucosa improved, but only when the amount of dye was not excessive, causing it to pool over the gastric mucosal surface. In cases with extensive IC dye accumulation, suction and flushing were necessary to remove some of the dye and improve mucosal texture recognition.

For image assessment, 142 IC-CE images were selected and sent to the external evaluator (S.M.) two months after the submission of NBI-CE images, to reduce the risk of bias. Again, the evaluator was blinded to the histologic findings and excluded four of the images from assessment, two used for anatomical orientation and two due to insufficient quality. Thus, 138 images were assessed according to the respective mucosal appearance with IC-CE and categorized as normal, suspicious, or abnormal. Table 5 summarizes the gastric mucosal patterns found in the IC-CE images and their corresponding histopathological diagnoses.

For the 138 IC-CE images, histological diagnoses were as follows: normal gastric mucosa (*n* = 46), inflammation of different type and degree (*n* = 50), mucous metaplasia (*n* = 1), glandular dysplasia (*n* = 23), and carcinoma (*n* = 18). The assessment of gastric IC-CE images as normal (negative) or suspicious/abnormal (positive) for pathological changes in histology had a sensitivity of 31%, specificity of 83%, and diagnostic accuracy of 67%.

## 4. Discussion

We found a higher diagnostic yield of biopsies taken during endoscopic procedures combining WLE with NBI-CE or IC-CE than from biopsies taken under WLE alone. The histology of both biopsy sets agreed in 60% of the dogs (18/30), but in other 30% (9/30) the gastric mucosal pathologies were more often diagnosed in targeted biopsies from WLE in combination with NBI-CE or IC-CE than in biopsies taken as indicated by WLE alone. The most prominent difference was that the diagnosis of gastric carcinoma was missed by biopsies taken under WLE. Independent from the biopsy set, histology revealed no mucosal changes in 13/30 dogs and changes in 17/30 dogs. Gastric mucosal structural changes were detected in 9/17 dogs (53%) using targeted biopsies guided by the combination of WLE and CE. In contrast, WLE biopsies found such histologic changes in only 2/17 dogs (12%). An agreement of mucosal structural pathology in both biopsy sets was seen in 6/17 dogs (35%). These results give some evidence that targeted biopsies guided by WLE in combination with CE can improve the diagnostic yield at histology, in comparison to WLE alone or non-targeted biopsies.

Chromoendoscopic procedures have gained wide application in human medicine but studies about their usefulness in veterinary medicine are scarce [6,13]. In people, improved image quality and light manipulation have led to major improvements in early gastric cancer diagnosis [24] and staging [25]. Our first objective was to assess whether real-time findings of WLE combined with two chromoendoscopic methods (NBI-CE and IC-CE) allow an improved biopsy sampling with a higher diagnostic yield at histology than using WLE alone. The second objective was to investigate whether templates used in humans for NBI-CE or IC-CE image assessments can predict gastric mucosal changes in dogs. However, the image assessment based on the NBI-CE template and expertise in IC-CE from human medicine revealed differing results. The estimated sensitivities, specificities, and accuracies for gastric mucosal changes of concern (gastric metaplasia, dysplasia, or carcinoma) showed that the human template is insufficient to match histologic confirmation or exclusion of gastric mucosal pathology in dogs. 

Several aspects need to be discussed that might have influenced the outcome of the study. These include the investigated dog population, the study design, and some limitations that need to be taken into consideration.

Gastric carcinoma accounts for only 1% of all neoplastic diseases in dogs, which means it is rather rare [26]. Belgian Shepherd dogs have been considered the best study population because especially the breed types Tervuren and Groenendael are highly predisposed not only for gastric carcinoma but also for mucous metaplasia and glandular dysplasia [27], both considered as preneoplastic changes of the gastric mucosa in humans and dogs [28]. Previous studies in humans used different prospective study designs comparing NBI-CE with dye-based CE (e.g., Lugol’s solution, methylene blue, IC) in the gastrointestinal tract (esophagus to colon). They included (1) a randomized order of endoscopy methods requiring two successive procedures in the same patient [29,30], (2) comparison of two groups of patients being randomly allocated to either NBI or dye-based CE [31,32], or (3) performing WLE followed by dye-based CE and NBI-CE, or NBI-CE followed by dye-based CE [33,34]. We decided to perform first WLE followed by NBI-CE and successively IC-CE for three reasons. First, pet dog owners would not consent otherwise, as there would be no ethical justification for two consecutive endoscopies just for research purposes. Second, we expected a case number too small to allow for the statistical comparison of two patient groups, one undergoing WLE and NBI-CE and the other WLE and IC-CE. Third, IC-CE was chosen to be the last procedure after NBI-CE to avoid lengthy washing of the gastric mucosa for dye removal and to prevent any interference of IC-CE with NBI-CE. This allowed for all three endoscopic procedures to be performed during one general anesthesia and obtaining endoscopic biopsies at the end of the procedure because these lead to microhemorrhage that could otherwise interfere with the CE results. One limitation of this approach is that the same endoscopist performed all three consecutive endoscopic examinations, which can cause a bias towards the assessment of the gastric mucosal patterns and the decision about sampling sites for the gastric mucosal biopsies. To address this bias, targeted biopsies were taken based on the findings of WLE combined with both CE methods, followed by biopsies that were non-targeted or directed by the initial records from WLE at the start of the procedure. In addition, NBI-CE and IC-CE images were sent two months apart to an external examiner (S.M.) who was blinded to both patient data and histologic diagnosis.

Our study showed an improved diagnostic yield when combining WLE with CE. This is consistent to some degree with data reported in human medicine. In one prospective study in humans, the diagnostic accuracy of WLE for extensive gastric intestinal metaplasia was 60% in a cohort of 25 patients. In comparison, the real-time diagnostic accuracy of high-resolution light-NBI in 35 patients was 87%. Those authors concluded that more than 90% of individuals at risk for gastric cancer could be identified without the need for biopsies [35]. In contrast, a recently published, open-label, randomized, controlled tandem trial in 4523 Japanese patients revealed that the overall sensitivity of primary endoscopy to detect early gastric cancer in humans was only 75% when using second-generation NBI-CE, therefore not increasing the detection rate over conventional WLE [36]. These conflicting experiences in human medicine should be taken into consideration when interpreting the results of our study. It may seem obvious that a definitive diagnosis of precancerous or early cancerous gastric mucosal pathology cannot be done without histology. However, improved visualization of changed gastric mucosal patterns by adding NBI-CE or IC-CE to WLE can lead to improvement by directing biopsies to target the areas with gastric mucosal structural changes. The addition of CE methods to WLE might minimize the risk of inadvertently sampling from areas without gastric mucosal pathology, thus achieving an inaccurate diagnosis with possibly detrimental consequences for the affected dog.

In 3/6 dogs, the carcinoma was undiagnosed in the biopsy set 2, referring to WLE-directed sampling. Although two of those dogs had very subtle, initial lesions (dogs 1 and 29), another case (dog 15) was diagnosed with a mass-like ulcerated carcinoma. The most plausible reason for the failure to diagnose the advanced case could be explained by insufficient sampling, missing the actual tumor. It is known that the diagnosis of gastric carcinoma sometimes requires full-thickness biopsies [1] as the invasive tumor cells are often found only deeper into the gastric wall, under the superficial mucosal layer. For the more subtle, early cases, it might have been expected that CE could facilitate selecting suspected biopsy sites, but there can also be other causes for the misdiagnosis. The WLE biopsy set 2 was collected last when blood from the previous sampling could have impaired exact visualization of the intended site, which could have been relevant with small changes.

Some issues affecting image quality and assessment are worthy of mention. We used the first-generation, 180-series NBI technology, missing the magnification function that can be enabled in the latest, more costly NBI equipment. In general, that restricted the ability to achieve ideal focus and final resolution of the still images submitted for assessment, which were paired with the histopathological diagnoses. The exact biopsy site was therefore considerably smaller than the area of the image assessed. On the other hand, the intensity of the light was often insufficient during real-time endoscopic examination when NBI function was on, which only allowed the adequate examination of restricted areas at a time, at close-up, and yet with limited resolution. In addition, the regular (white) light was insufficient to assess the mucosa after it had been sprayed with the dark-blue IC.

CE requires excellent mucosal preparation and image quality was affected by the smallest amounts of food and debris (soil, hair) even after adequate fasting. Similarly, despite the mucolytic and antifoaming agents administered to improve mucosal preparation, remaining strains of mucus or abundant fluid and foam (saliva) often required extensive flushing using the irrigation pump and cycles of suction to allow for detailed visualization. Even small amounts of bile-tinged duodenal mucus were a nuisance, as bile shows a misleading reddish hue in NBI-CE. Also, blood from ulcerated sites not only interfered with local mucosal imaging but also impaired the IC-CE staining. During the biopsy sampling steps, the combination of the dark-blue IC and bleeding from biopsy sites, with the limited illumination provided by the equipment, often required additional wash steps to carry on with the extensive sampling protocol.

In our experience, IC-CE does improve the perception of mucosal topography and texture, making it easier to identify suspected areas even without a close-up view during the initial evaluation of the stomach. Dye-spraying is much more affordable than NBI technologies, requiring only simple equipment and a couple of minutes to apply. It effectively enhances the texture of what is already present with WLE, grossly and superficially. On the other hand, NBI has the potential to show more subtle, inconspicuous, and deeper mucosal details. 

Our findings suggest that CE in dogs has the potential to improve the diagnosis of precancerous gastric mucosal pathology and early gastric carcinoma. However, the differences between the gastric mucosa of humans and dogs are such that a specific template must be developed for clinical application in canines. Future research should apply next-generation NBI equipment enabling magnification (e.g., Olympus 190/200 series) in search of early changes related to gastric carcinoma. Tervuren and Groenendael Belgian Shepherd dogs aged between five and six years, with the highest risk to be positive for precancerous structural changes of the gastric mucosa such as mucous metaplasia and glandular dysplasia [6,26], should be targeted in the next phase of the study, with focal imaging in exact correspondence with targeted biopsy sites.

## Figures and Tables

**Table 1:** Examples of mucosal pattern categories recognized in narrow band imaging chromoendoscopy (NBI-CE) of the gastric mucosa of dogs.

Gastric Mucosal Pattern Category	NBI-CE Example Image
Normal round	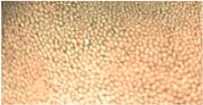
Normal mosaic and polygonal	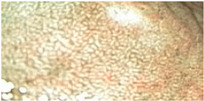
Enlarged mosaic and polygonal	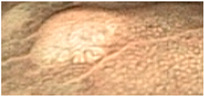
Tubulo-villous	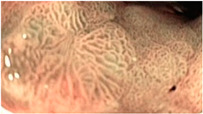

**Table 2:** Examples of mucosal categories recognized in indigo carmine chromoendoscopy (IC-CE) of the gastric mucosa of dogs.

Gastric Mucosal Pattern Category	IC-CE Example Image
Normal	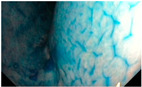
Suspicious	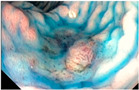

**Table 3:** Signalment of 30 Belgian shepherd dogs, findings during endoscopy, and histology from gastric mucosal biopsies: (1) sampling directed by white-light endoscopy (WLE) followed by chromoendoscopy (CE), and (2) biopsies taken as indicated by WLE alone.

Dog Nr.	Dog’s Study ID	Breed Type	Age in Years	Sex	Body Weight (kg)	Distribution of the Most Relevant Endoscopic Finding	Type of Inflammation	Mucous Metaplasia		Glandular Dysplasia		Carcinoma	
								Biopsy Set 1	Biopsy Set 2	Biopsy Set 1	Biopsy Set 2	Biopsy Set 1	Biopsy Set 2
1	641	Tervuren	10.8	M	26.1	Diffuse Abnormal folding surrounded by irregular mucosal surface in corpus: distal greater curvature	LP	**Yes**	**Yes**	**Yes**	No	**Yes**	No
2	642	Tervuren	9.9	M	22.7	Diffuse Very prominent blood vessels in proximal antrum; abnormal folding/ thicker mucosa in corpus: distal greater curvature	LP, EO, NP	No	No	No	No	No	No
3	644	Tervuren	10.1	FS	18.5	Diffuse Major blood vessels visible in most of corpus, especially along greater curvature; marked mucosal folding in pylorus	LP, EO, NP	No	No	No	No	No	No
4	645	Tervuren	11.6	FS	25.3	Focal Sessile polyp 2 mm × 5 mm	LP, EO, NP	No	No	No	No	No	No
5	646	Groenendael	11.2	M	23.0	Diffuse Persistent folds after full insufflation in corpus: distal greater curvature	LP, NP	No	No	No	**Yes**	No	No
6	647	Tervuren	10.1	FS	23.4	Diffuse Abnormal folding in corpus: distal greater curvature	LP, NP	**Yes**	**Yes**	**Yes**	**Yes**	**Yes**	**Yes**
7	649	Tervuren	7.9	F	20.5	Diffuse Irregular mucosal surface in corpus: distal greater curvature	LP, EO, NP	No	No	No	No	No	No
8	650	Groenendael	10.6	M	23.0	Unremarkable	LP, NP	No	No	No	No	No	No
9	702	Malinois	11.1	MC	31.6	Focal Irregular mucosal surface in corpus: distal greater curvature	LP, EO	**Yes**	No	No	No	No	No
10	703	Tervuren	9.0	M	21.3	Diffuse More than 20 discolored patches 5 mm all over antrum	LP, EO	No	No	No	**Yes**	No	No
11	652	Tervuren	10.1	MC	30.7	Ulcerative More than 30 erosions 2 mm in proximal greater curvature	LP, EO	No	No *	**Yes**	No	No	No
12	653	Malinois	6.7	FS	36.6	Diffuse Hyperemic spots around cardia (lymphoid follicles)	LP, EO	No	No	No	No	No	No
13	654	Tervuren	7.6	M	29.3	Ulcerative Linear ulcers in corpus: distal lesser curvature	LP, EO, NP	No	No	No	No	No	No
14	655	Groenendael	9.1	FS	22.2	Diffuse Hyperemic spots around cardia	LP, EO	No	No	No	No	No	No
15	658	Tervuren	7.2	M	27.8	Ulcerative Massive elevation in mucosa, two large crater-like ulcers in corpus	LP, EO, NP	**Yes**	No	**Yes**	No	**Yes**	No
16	664	Tervuren	10.4	FS	22.6	Focal Tiny polyp in pylorus	LP, EO, NP	No	No	**Yes**	No	No	No
17	677	Groenendael	8.3	F	17.4	Ulcerative Long ulcer crater in corpus, closer to lesser curvature; gross (mural) thickening of the incisura	LP, EO, NP	No	No	**Yes**	**Yes**	**Yes**	**Yes**
18	689	Malinois	8.8	FS	22.2	Unremarkable	LP, EO, NP	No	No	**Yes**	**Yes**	No	No
19	687	Groenendael	9.7	FS	27.7	Unremarkable	LP, EO, NP	No	No	**Yes**	**Yes**	No	No
20	686	Tervuren	5.6	F	21.5	Unremarkable	LP, EO, NP	No	No	No	No	No	No
21	679	Tervuren	9.0	FS	17.1	Diffuse Visible blood vessels on gastric body	LP, EO, NP	No	No	No	No	No	No
22	722	Tervuren	11.2	M	29.8	Diffuse Greater curvature: persisting folds in distal corpus, irregular surface in antrum	LP, EO, NP	**Yes**	**Yes**	**Yes**	No	No	No
23	721	Tervuren	11.2	FS	23.2	Ulcerative Three small ulcers along lesser curvature and thickened mucosa in distal greater curvature of corpus; hyperemic antrum greater curvature	LP, EO, NP	No	**Yes**	**Yes**	**Yes**	**Yes**	**Yes**
24	680	Tervuren	5.5	F	22.7	Diffuse Distal greater curvature of corpus: very marked persistent folding with irregular surface and multifocal hyperemia, linear depression	LP, NP	**Yes**	No	**Yes**	**Yes**	No	No
25	750	Tervuren	10.1	FS	25.5	Diffuse Corncob texture along the left side of the corpus	LP, EO	**Yes**	No	**Yes**	**Yes**	No	No
26	749	Groenendael	8.3	MC	27.2	Unremarkable	LP	No	No	No	No	No	No
27	747	Tervuren	8.2	F	29.5	Unremarkable	LP, EO	**Yes**	**Yes**	No	No	No	No
28	724	Groenendael	7.6	F	24.9	Diffuse Corpus: elevated mucosa with multifocal, depressed darker spots along proximal greater curvature; visible small vessels in distal lesser curvature	LP, EO	No	No	No	No	No	No
29	711	Groenendael	8.3	FS	24.6	Ulcerative Elongated, small transversal, non-bleeding crater in transition between corpus and antrum, left of greater curvature	LP, EO	No	No	**Yes**	No	**Yes**	No
30	719	Tervuren	11.3	FS	18.2	Unremarkable	LP, EO	No	No	No	No	No	No

Legend: M—male; MC—male castrated; F—female, FS—female spayed; LP—lymphoplasmacytic; EO—eosinophilic; NP—neutrophilic infiltrations in lamina propria. Histologies that differ between the two biopsy sets are highlighted in a gray background, the presence of changes of concern are highlighted in bold. * Intestinal metaplasia (AB-PAS positive).

**Table 4:** Most severe histopathological diagnoses of biopsy sites and categories of gastric mucosal patterns of dogs identified in 87 endoscopic still images obtained during NBI-CE. 4.1. 2 × 2 table for histopathological diagnosis and NBI-CE mucosal patterns.

Histopathological Diagnosis	Type of Mucosal Pattern at NBI-CE			
	Normal		Abnormal	
	Round (*n* = 44)	Mosaic and Polygonal (*n* = 21)	Enlarged Mosaic and Polygonal (*n* = 12)	Tubulo-Villous (*n* = 10)
Normal (*n* = 24)	14	7	3	0
Inflammation (*n* = 32)	19	6	4	3
Mucous metaplasia (*n* = 2)	1	1	0	0
Glandular dysplasia (*n* = 16)	8	4	4	0
Carcinoma (*n* = 13)	2	3	1	7
**(4.1)**				
**NBI-CE Mucosal Pattern**	**Histopathological Diagnosis**			
	**Normal/Inflammation (*n* = 56)**		**Meta-/Dysplasia/Carcinoma (*n* = 31)**	
Normal (*n* = 65)	46		19	
Abnormal (*n* = 22)	10		12	

**Table 5:** Most severe histopathological diagnosis of biopsy sites and categories of gastric mucosal assessment of dogs identified in 138 endoscopic still images taken during IC-CE. 5.1. 2 × 2 table for histopathological diagnosis and IC-CE mucosal assessment.

Histopathological Diagnosis	IC-CE Mucosal Appearance			
	Normal (*n* = 109)	Suspicious (*n* = 20)		Abnormal (*n* = 9)
Normal (*n* = 46)	40	6		0
Inflammation (*n* = 50)	40	8		2
Mucous metaplasia (*n* = 1)	1	0		0
Glandular dysplasia (*n* = 23)	20	2		1
Carcinoma (*n* = 18)	8	4		6
**(5.1)**				
**IC-CE Mucosal Appearance**	**Histopathological Diagnosis**			
	**Normal/Inflammation (*n* = 96)**		**Meta-/Dysplasia/Carcinoma (*n* = 42)**	
Normal (*n* = 109)	80		29	
Suspicious/Abnormal (*n* = 29)	16		13	

## Data Availability

Not applicable.
